# Characteristics of lower respiratory tract pathogens in critically ill patients with heatstroke: a retrospective multi-center study

**DOI:** 10.3389/fcimb.2024.1420535

**Published:** 2024-11-28

**Authors:** Aijia Ma, Xinyang Jin, Yucong Wang, Lietao Wang, Lvyuan Shi, Dingyuan Wan, Qin Wu, Min He, Zhuo Tang, Jiajin Li, Jian Wang, Guangwei Yang, Zhongwei Zhang, Jing Yang, Bo Wang

**Affiliations:** ^1^ Department of Critical Care Medicine, West China Hospital of Sichuan University, Chengdu, Sichuan, China; ^2^ School of Pharmacy, Macau University of Science and Technology, Macao, Macao SAR, China; ^3^ Department of Intensive Care Medicine, Zizhong County Chinese Medicine Hospital, Zizhong, Sichuan, China; ^4^ Department of Intensive Care Medicine, Mianyang People’s Hospital, Mianyang, Sichuan, China; ^5^ Department of Intensive Care Medicine, Guang’an People’s Hospital, Guang’an, Sichuan, China; ^6^ Department of Intensive Care Medicine, Huaying People’s Hospital, Huaying, Sichuan, China

**Keywords:** heatstroke, lower respiratory tract infections, high body temperature, lung pathogen, intensive care unit

## Abstract

This retrospective multicenter study was conducted across 83 intensive care units (ICUs) in 16 cities in Sichuan, China. Critically ill patients diagnosed with heatstroke and lung infections were included in the study. Specimens from the lower respiratory tract were collected for microbiological testing, and the characteristics of the pathogens were described. A total of 462 patients diagnosed with heatstroke-related pulmonary infections were included, 134 patients (29.0%) tested positive for respiratory pathogens. The most frequently isolated strain was *Klebsiella pneumoniae* (34.3%), followed by *Escherichia coli* (28.4%), *Staphylococcus aureus* (20.9%). The results revealed that in the hyperthermic resistance group, there was a higher proportion of *Pseudomonas aeruginosa* [14(34.1%) vs 11(11.8%), *p*=0.002] and *Stenotrophomonas maltophilia* [4(9.8%) vs 1(1.1%), *p*=0.030] compared to the hyperthermic control group. And a higher proportion of *Staphylococcus aureus* [27(29.7%) vs 1(2.3%), *p*<0.001], were obtained during the earlier stages with elevated temperatures. Patients with *Klebsiella pneumoniae* (38.3 ± 1.9°C), *Staphylococcus aureus* (38.5 ± 2.2°C), and *Pseudomonas aeruginosa* (38.7 ± 1.9°C) exhibited a higher temperature environment. Our study provides crucial insights into the lower respiratory tract pathogenesis of heatstroke patients, identifying key pathogens and their temperature-dependent characteristics, thus providing a foundation for future empirical treatment strategies in heatstroke.

## Introduction

With the intensification of global climate extremes, the radiating heat wave has now encroached upon regions that were previously spared from its scorching touch (Tuholske et al., 2021; Watts et al., 2021). A daunting reality emerges as an increasing number of individuals are confronted with laboring under unrelenting heat, rendering them vulnerable to the severe heatstroke in the absence of precautionary measures (Périard et al., 2022). Heatstroke, a highly fatal disease, arises from imbalanced heat generation and dissipation due to exposure to hot environments or strenuous exercise, and is characterized by multiple organ failure and extreme hyperthermia (core body temperature above 40°C) (Epstein and Yanovich, 2019; Bouchama et al., 2022).

Situated in the southwestern region of mainland China, Sichuan Province is characterized by its unique basin topography (Feng et al., 2020). In 2022, the Sichuan Basin was subjected to a confluence of subtropical and sub-tropical high-pressure systems, resulting in an unprecedented surge in temperatures, reaching historic highs not witnessed since 1961. The scorching heatwave of 2022 was predicted likely just the beginning for the next decade (Chen et al., 2022; Coleman, 2022). The increasing number of critically ill heatstroke patients in ICU serves as a clear indication that it is time to prepare in advance for this climate battle.

It is widely acknowledged that living organisms exhibit temperature sensitivity, whereby alterations in environmental temperature and thermal stress can profoundly impact microbial survival and colonization across diverse organ systems (Hylander and Repasky, 2019). Notably, prior investigations have unveiled an association between core temperature fluctuations and changes in the composition and functionality of the human microbiome, revealing the capacity of certain microorganisms to adapt beyond their optimal thermal thresholds (Huus and Ley, 2021). Within the realm of severe heatstroke, the pulmonary microbiome is significantly importance, given its pivotal role as a primary hit target of heatstroke (Patel et al., 2023). However, a crucial knowledge gap persists concerning the potential alterations in the lung microbiome at the baseline of elevated core temperature in patients with heatstroke. These alterations may lead to variations in the pathogenesis of pulmonary infections and have implications for antibiotic utilization.

The incidence of heatstroke is progressively increasing, leading to a rising number of critically ill patients requiring admission to the intensive care unit. However, the precise impact of temperature on the occurrence of pulmonary infections and the underlying pathogenesis remains poorly understood. Hence, the primary aim of this study is to elucidate the impact of high core body temperature in heatstroke patients on the diversity of detected pulmonary pathogens, providing invaluable guidance to clinicians in the timely and empirical use of antibiotics for early intervention in heatstroke-related pulmonary infections.

## Materials and methods

### Setting and study design

This retrospective multicenter study was conducted in 83 ICUs located in 16 cities across Sichuan, China. This study aimed to investigate the clinical epidemiological characteristics and lung pathogenic features of severe heatstroke during the heatwave in the Sichuan Basin in 2022. The study was reviewed and approved by the Biomedical Ethics Review Committee of West China Hospital of Sichuan University (approval number: SCU-2022-1542, registration number: ChiCTR2200066314).

The inclusion criteria for this study were as follows: (1) patients aged >18 years, (2) patients admitted to ICU due to heatstroke and heatstroke-related complications, and (3) patients diagnosed with lung infection.

Heatstroke is defined as a condition in which a patient has a history of exposure to high temperatures and humidity, extreme heat (core body temperature >40.5°C) on admission to the emergency department, and presents with consciousness disturbances, respiratory difficulties, hypotensive shock (mean arterial pressure <65 mmHg), and various complications such as arrhythmia, liver and kidney dysfunction, coagulation disorders, and significant fluid loss (3,000–8,000 ml) before ICU admission (Epstein and Yanovich, 2019; Bouchama et al., 2022).

Lung infection was defined based on a combination of radiological, clinical, and pathogenic positive (Jaroszewski et al., 2012). Radiological evidence included the presence of new or aggravated infiltration observed on chest radiograph or CT scans. Clinical evidence involved signs and symptoms indicative of an infection. Pathogenetic confirmation came from clinical reports reviewed by clinicians in the clinical laboratory of each hospital. No major epidemiological events occurred during the study period.

### Sample procedures

Samples for pathogenic examination were collected from the lower respiratory tract, including sputum, bronchoalveolar lavage fluid (BALF), and endotracheal aspirate. Samples were first collected on the day of ICU admission and every 2-3 days thereafter. Endotracheal aspirate was specifically obtained from patients receiving invasive mechanical support, such as tracheal intubation or tracheotomy.

BALF was obtained by a clinician performing bronchoalveolar lavage. The first tube of BALF was discarded during lavage, and specimens for pathogenetic analysis were collected in sterile containers. Each patient is required to collect 10-15 ml of alveolar lavage fluid for pathogenetic testing. Microbiological smears and cultures were performed by trained professionals at the medical testing centers of the respective hospitals, following standard microbiological methods. The standard incubation period for bacteria is typically 48 to 72 hours, although this may vary depending on the specific pathogen and culture conditions. Polymerase Chain Reaction (PCR) methods were not employed in this study.

### Statistical analysis

Statistical analysis was performed using SPSS version 22.0 and GraphPad 8.0. Categorical variables were analyzed using the Chi-square test, while continuous variables were compared using the t-test between two groups. A *p*-value of less than 0.05 was considered statistically significant in all comparisons.

## Result

### Patient demographics and clinical characteristics

Within our cohort of 873 patients primarily admitted to the ICU for heatstroke during June, 2022 and October, 2022, a total of 462 patients were diagnosed with lung infection, resulting in an incidence rate of 52.9%.

Specifically, among the 462 patients who developed lung infections, 134 individuals (29.0%) had pathogenic organisms positively isolated from lower respiratory tract specimens, while the remaining 328 cases (71.0%) included pathogenically negative as well as empirical diagnoses based on clinical experience. **Table 1** presents the clinical characteristics of these 462 patients. Patients susceptible to heatstroke were characterized by advanced age (71.5 ± 13.9), predominantly male gender (n=265, 57.4%), and a range of underlying diseases, including hypertension (n=121, 26.2%), chronic obstructive pulmonary disease (n=95, 20.6%), chronic cardiac insufficiency (n=55, 11.9%), and diabetes mellitus (n=65, 14.1%).

**Table 1:** Clinical characteristics of heatstroke patients diagnosed with lung infection.

	All (n=462)	Positive pathogen(n=134)	Negative pathogen (n=328)	*p*
Age, year	71.5 ± 13.9	69.7 ± 15.9	72.2 ± 13.0	0.108
Male, n (%)	265 (57.4)	79 (59.0)	186 (56.7)	0.658
BMI, kg/m2	22.2 ± 2.8	21.7 ± 2.7	22.4 ± 2.8	0.012*
CRP, mg/L	24.3 ± 43.5	36.3 ± 41.0	20.2 ± 43.6	0.019*
GCS score	6.9 ± 4.4	5.9 ± 3.3	7.2 ± 4.6	0.003*
PCT, ug/L	17.2 ± 49.5	19.8 ± 26.4	16.2 ± 56.2	0.544
WBC, 10^9/L	12.6 ± 5.8)	12.2 ± 5.3	12.8 ± 6.0	0.364
PaO2/FiO2 ratio	258.2 ± 138.9	255.9 ± 135.0	259.0 ± 140.4	0.839
Hyperthermic resistance, n (%)	96 (20.8)	41 (30.6)	55 (16.8)	0.001*
ICU first temperature, °C	40.6 ± 1.1	40.5 ± 1.1	40.6 ± 1.1	0.539
ICU 48h temperature, °C	37.5 ± 1.6	37.8 ± 1.6	37.3 ± 1.0	0.002*
Heat Stroke Classification, n (%)				
Labor	93 (20.1)	29 (21.6)	64 (19.5)	0.604
Classic	316 (68.4)	77 (57.5)	239 (72.9)	<0.001*
Unclear	53 (11.5)	28 (20.9)	25 (7.6)	<0.001*
Underlying Diseases, n (%)				
Chronic liver dysfunction	10 (2.2)	3 (2.2)	7 (2.1)	0.944
Hypertension	121 (26.2)	34 (25.4)	87 (26.5)	0.798
Rheumatic immune system diseases	8 (1.7)	4 (3.0)	4 (1.2)	0.238
Chronic cardiac insufficiency	55 (11.9)	9 (6.7)	46 (14.0)	0.028*
Chronic obstructive pulmonary disease	95 (20.6)	24 (17.9)	71 (21.6)	0.367
Chronic renal dysfunction	13 (2.8)	3 (2.2)	10 (3.0)	0.633
Diabetes	65 (14.1)	20 (14.9)	45 (13.7)	0.735
Complication, n (%)				
Water-electrolyte disorders	284 (61.5)	79 (59.0)	205 (62.5)	0.477
Central nervous system damage	183 (39.6)	65 (48.5)	118 (36.0)	0.012*
Myocardial damage	200 (43.3)	68 (50.7)	132 (40.2)	0.018*
Transverse rhabdomyolysis	79 (17.1)	33 (24.6)	46 (14.0)	0.004*
Renal impairment	202 (43.7)	63 (47.0)	139 (42.4)	0.275
Coagulation disorders	155 (33.5)	49 (36.6)	106 (32.3)	0.318
Respiratory failure	185 (40.0)	67 (50.0)	118 (36.0)	0.002*
Gastrointestinal bleeding	54 (11.7)	20 (14.9)	34 (10.4)	0.15
Liver impairment	216 (46.8)	68 (50.7)	148 (45.1)	0.185
Combined infection, n (%)				
With bloodstream infection	11 (2.4)	8 (6.0)	3 (0.9)	0.003*
With urinary tract infection	9 (1.9)	7 (5.2)	2 (0.6)	0.001*
With skin and soft tissue infections	7 (1.5)	3 (2.2)	4 (1.2)	0.713
Respiratory support, n (%)				
Invasive ventilator	295 (63.9)	109 (81.3)	186 (56.7)	<0.001*
Non-invasive or high flow	10 (2.2)	5 (3.7)	5 (1.5)	0.139
Nasal catheter or mask	157 (34.0)	20 (14.9)	137 (41.8)	<0.001*
Clinical outcome, n (%)				
Dead	109 (23.6)	35 (26.1)	74 (22.5)	0.414
Transferred out	29 (6.3)	16 (11.9)	13 (4.0)	0.001*
Transferred with tracheal intubation	9 (1.9)	3 (2.2)	6 (1.8)	0.723

Data are presented as n (%) and mean ± SD.

SD, standard deviation; ICU, intensive care unit; CRP, c reactive protein; PCT, procalcitonin; WBC, white blood cell; BMI, body mass index; GCS, glasgow coma scale.

*p < 0.05 was considered statistically significant.

During the hospitalization period, the included cohort of 462 heatstroke patients also experienced life-threatening complications, including: water-electrolyte disturbances (n=284, 61.5%), liver impairment (n=216, 46.8%), renal impairment (n=202, 43.7%), myocardial impairment (n=200, 43.3%), respiratory failure (n=185, 40.0%), coagulation disorders (n=155, 33.5%), and gastrointestinal bleeding (n=54, 11.7%). Invasive mechanical ventilation was required for respiratory support in 295 patients (63.9%), with a significantly higher intubation rate observed in patients with positive pathogenicity compared to those with negative pathogenicity [109 (81.3%) vs 186 (56.7%), *p*<0.001]. Among the 462 heatstroke patients diagnosed with lung infection, a total of 109 individuals (23.6%) experienced mortality, while 29 patients (6.3%) required transfer to other medical facilities, including 9 cases (31.0%) necessitating tracheal intubation.

Effective temperature monitoring in patients with heatstroke is imperative. Upon admission to the ICU, the maximum recorded temperature was found to be 40.6 (1.1) °C. There was no significant different in the maximum temperature at ICU admission between patients with positive and negative lung infections [40.5 (1.1%) vs 40.6 (1.1%), *p*=0.539]. However, after 48 hours of receiving cooling measures in the ICU, the pathogenically positive group exhibited higher temperatures compared to the pathogenically negative group [37.8 (1.6%) vs 37.3 (1.0%), *p*=0.002], which indicated that the presence of positive pathogens continued to impact the body temperature of patients with heatstroke.

### Characteristics of the pathogen isolated from the lower respiratory tract in heatstroke

We further investigated the characteristics and differences of lower respiratory tract pathogenesis in patients with heatstroke, aiming to provide valuable insights for improved empirical antibiotic treatment in the future.

**Figure 1:**
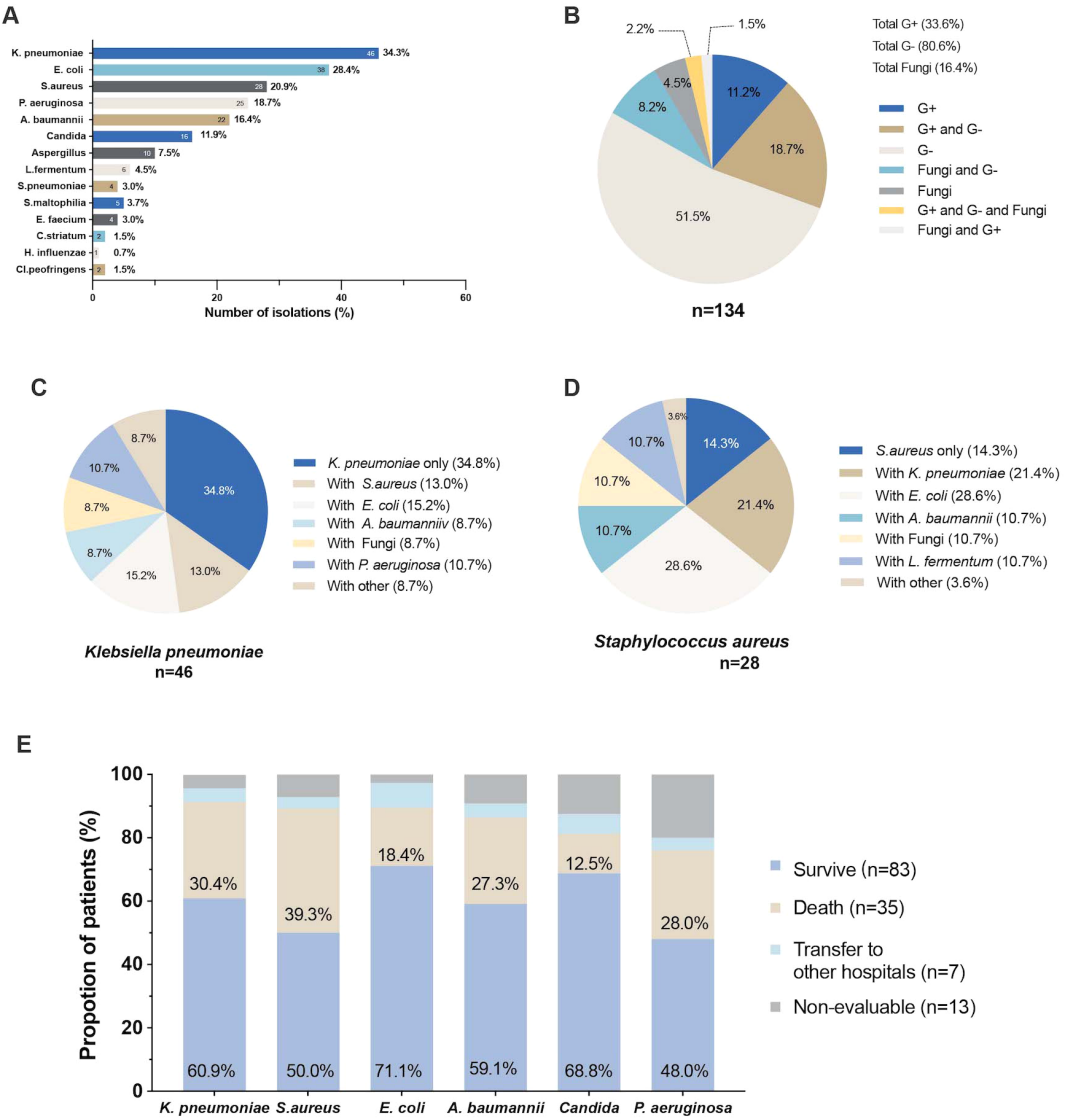
The pathogen isolated from the lower respiratory tract in heatstroke **(A)** Total number of pathogens isolated from the lower respiratory tract; **(B)** Pie chart of the percentage of isolated Gram-positive, Gram-negative and fungi in lower respiratory tract; **(C)** Pie chart of the percentage of co-isolation of *Klebsiella pneumoniae* with other pathogens; **(D)** Pie chart of the percentage of co-isolation of *Staphylococcus aureus* with other pathogens; **(E)** Proportion of clinical outcomes in patients with different pathogenesis.

Among the 134 patients, pathogenic strains were isolated from lower respiratory tract specimens (**Figure 1A**), with a predominance of Gram-negative strains (n=108, 80.6%), followed by Gram-positive strains (n=45, 33.6%), and fungi (n=22, 16.4%). Patients frequently exhibited isolation of multiple bacterial strains, with a high proportion (18.7%) demonstrating coexistence of Gram-positive and Gram-negative bacteria. Additionally, Gram-negative bacteria were commonly isolated alongside fungi (8.2%), while the isolation of Gram-positive bacteria, Gram-negative bacteria, and fungi occurred at a probability of 2.2% (**Figure 1B**).

In general, the most frequently isolated strain was *Klebsiella pneumoniae* (n=46, 34.3%), followed by *Escherichia coli* (n=38, 28.4%), *Staphylococcus aureus* (n=28, 20.9%), *Pseudomonas aeruginosa* (n=25, 18.7%), *Acinetobacter baumannii* (n=22, 16.4%), *Candida* (n=16, 11.9%), *Aspergillus* (n=10, 7.5%), *Lactobacillus fermentum* (n=6, 4.5%), and *Streptococcus pneumoniae* (n=4, 3.0%) (**Figure 1A**).

Among patients who isolated *Klebsiella pneumoniae* strains, the most common co-isolation was with *Escherichia coli* (15.2%), followed by *Staphylococcus aureus* (13.0%), while only 8.7% of patients exhibited co-isolation with fungi. Similarly, patients who isolated *Staphylococcus aureus* strains often had concomitant *Escherichia coli* (28.6%), *Klebsiella pneumoniae* (28.4%), and *Lactobacillus fermentum* (10.7%) strains (**Figures 1C, D**). The isolation of different bacterial strains has been shown to have varying impacts on prognosis. Among the isolated strains, patients with *Staphylococcus aureus* had the highest mortality rate (39.3%), followed by those with *Klebsiella pneumoniae* (30.4%), *Pseudomonas aeruginosa* (28.0%), and *Acinetobacter baumannii* (27.3%) (**Figure 1E**).

### Relationship between temperature and pathogen in patients with heatstroke

**Figure 2:**
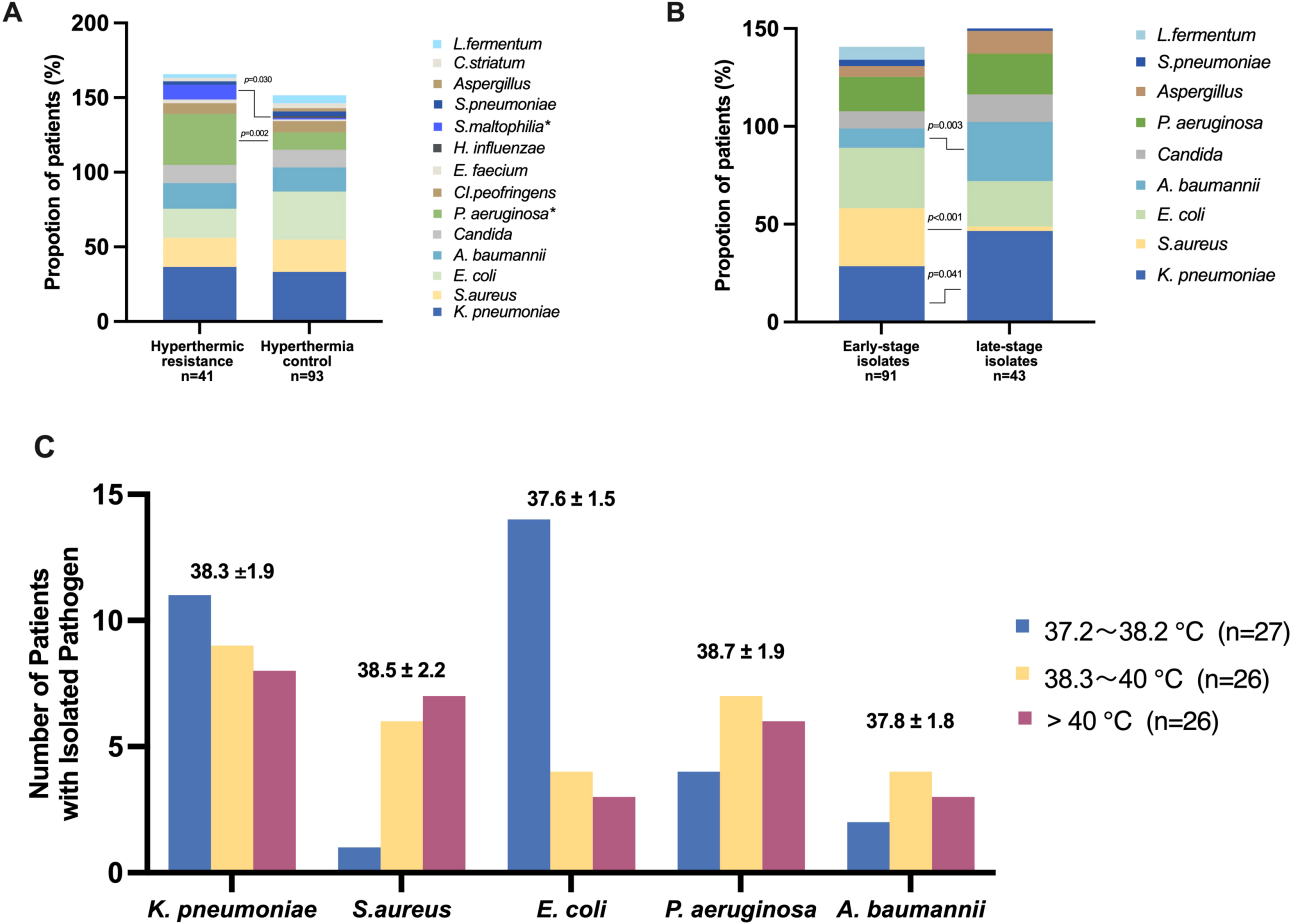
Relationship between temperature and pathogen in patients with heatstroke **(A)** Percentage of different pathogens in the two groups of hyperthermic resistance (patients remained high temperature (>38°C) 48 hours after admission to the ICU) and hyperthermic control (patients responded well to cooling); **(B)** Percentage of different pathogens in the two groups of early-stage isolates(patients with strains isolated within 48 hours of admission to the ICU) and late-stage isolates(patients with strains isolated after 48 hours of admission to the ICU); **(C)** Number of patients with isolated pathogen in different temperature intervals. The temperature of patients isolated with different pathogens was expressed as mean ± standard deviation.

We observed that in some heatstroke patients, despite the implementation of cooling measures, achieving a body temperature below 38°C remains challenging. We hypothesized that the respiratory microbiota in these individuals may differ due to prolonged exposure to high temperatures. To investigate this further, we selected patients who remained high temperature (>38°C) 48 hours after admission to the ICU from the cohort of 134 patients with positive pathogenic findings. Out of the total 134 patients, 41 were included in this subgroup, referred to as the “hyperthermic resistance” group (**Figure 2A**). We observed distinct differences in the lower respiratory tract pathogen between the hyperthermic resistance group and the group that responded well to cooling, referred to as the “hyperthermia control” group. The results revealed that in the hyperthermic resistance group, there was a higher proportion of *Pseudomonas aeruginosa* [14(34.1%) vs 11(11.8%), *p*=0.002] and *Stenotrophomonas maltophilia* [4(9.8%) vs 1(1.1%), *p*=0.030] compared to the hyperthermic control group. However, there were no significant differences in the proportions of *Klebsiella pneumoniae* [15(36.6%) vs 31(33.3%), *p*=0.715], *Staphylococcus aureus* [8(19.5%) vs 20(21.5%), *p*=0.794], and *Acinetobacter baumannii* [7(17.1%) vs 15(16.1%), *p*=0.892] between the two groups. Escherichia coli exhibited a relatively high proportion in both groups, with a trend towards higher proportions in the hyperthermic control group [8(19.5%) vs 30(32.3%), *p*=0.131] (**Figure 2A**).

Furthermore, the timing of strain isolation may have prognostic implications for heatstroke patients. We specifically examined patients with strains isolated within 48 hours of their admission to the ICU. Patients who experienced higher temperatures during the early stages of heatstroke might possess a distinct respiratory microbiome compared to those with strains screened after 48 hours, during which most patients have undergone cooling interventions. Among the 91 patients with pathogenic strains isolated within 48 hours of ICU admission, we analyzed the baseline characteristics of two groups: the early-stage isolation group and the late-stage isolation group. Notably, there were no significant differences observed in the maximum temperature recorded at the time of ICU admission [40.5(1.1%) vs 40.7(1.1%), *p*=0.473], nor in the 48-hour temperature [37.9(1.1%) vs 37.6(1.1%), *p*=0.730] between the two groups. However, a significant disparity was noted in the body temperature at the time of strain isolation, with the early isolates associated with higher body temperatures compared to the late isolates [38.6(2.0%) vs 37.7(1.6%), *p*=0.006]. Furthermore, patients with early ICU isolates exhibited a higher mortality trend [28(30.8%) vs 7(16.3%), *p*=0.075].

We conducted a comparison of the lower respiratory tract pathogenesis between the two groups (**Figure 2B**). The results indicated a higher proportion of positive isolates, particularly *Staphylococcus aureus* [27(29.7%) vs 1(2.3%), *p*<0.001], were obtained during the earlier stages with elevated temperatures. Conversely, negative bacteria were more frequently isolated during the late stage, specifically *Klebsiella pneumoniae* [26 (28.6%) vs 20 (46.5%), *p*=0.041], and *Acinetobacter baumannii* [9(9.9%) vs 13(30.2%), *p*=0.003]. There was no significant isolation of *Streptococcus pneumoniae* from the lower respiratory tract specimens in either the early- or late- stages isolates of heatstroke patients. *Lactobacillus fermentum* was predominantly isolated from early-stage isolates (6.6%) and was rarely detected in late-stage isolates.

Furthermore, we investigated the distribution of various bacterial strains based on the temperature ranges at the time of pathogen detection. Initially, we categorized the patients’ temperatures into five intervals: <36.5°C, 36.5-37.1°C, 37.2-38.2°C, 38.3-40°C, and >40°C. We conducted an analysis of the temperature intervals associated with fever, as depicted in **Figure 2C**. The results revealed that patients with *Klebsiella pneumoniae* (38.3 ± 1.9°C), *Staphylococcus aureus* (38.5 ± 2.2°C), and *Pseudomonas aeruginosa* (38.7 ± 1.9°C) exhibited higher temperature environments upon strain isolation. However, there was a decreasing trend in the number of *Klebsiella pneumoniae* and *Escherichia coli* isolations as the temperature increased, while *Staphylococcus aureus* showed an increasing trend with rising temperatures.

## Discussion

Our study represents a groundbreaking investigation into the lower respiratory tract pathogen characteristics of patients with heatstroke. The results revealed that *Klebsiella pneumoniae, Escherichia coli, Staphylococcus aureus*, and *Pseudomonas aeruginosa* are the primary pathogens in the lower respiratory tract of heatstroke patients with pulmonary infections. Notably, *Pseudomonas aeruginosa* exhibited a higher prevalence in hyperthermic resistance patients and was significantly associated with difficulties in temperature reduction among heatstroke patients. Despite the abundant presence of *Klebsiella pneumoniae* and *Escherichia coli*, their isolation decreased significantly when patients’ core temperatures exceeded 40°C. Furthermore, *Staphylococcus aureus* emerged as the predominant Gram-positive bacterium isolated from heatstroke patients with higher core temperatures, while the commonly encountered Gram-positive bacterium, *Streptococcus pneumoniae*, seemed unable to survive in the elevated temperatures of the lower respiratory tract. These findings highlight the pivotal significance of our study, providing crucial insights into the pathogenic microbiota and their temperature-dependent behavior in heatstroke patients. The significance of this study lies in our characterization of the pathogen features of pulmonary infections, which are commonly observed in heatstroke patients but remain poorly understood. By shedding light on the pathogenic profiles, we provide a foundation for future empirical treatment strategies, enhancing clinical management in the context of heatstroke.

Among critically ill patients with detectable pathogens, Gram-negative bacteria remain the predominant pathogens isolated from the lower respiratory tract of heatstroke patients, particularly *Klebsiella pneumoniae* and *Pseudomonas aeruginos*a *(* Folic et al., 2021; Ding et al., 2023). Simultaneously, high levels of Gram-positive bacteria, such as *Staphylococcus aureus*, were isolated from heatstroke patients, aligning with the prevailing patterns observed in the lower respiratory tract pathogenesis of ICU patients (Niven and Laupland, 2016; Paling et al., 2020). However, a notable finding was the significantly higher prevalence of *Escherichia coli* isolates in the lower respiratory tract microbiota of heatstroke patients, which were primarily associated with the co-occurrence of *Klebsiella pneumoniae* and *Staphylococcus aureus*. Interestingly, *Escherichia coli* were predominantly abundant in the temperature range of 37.2-38.2°C, as the temperature increased above 38.3 °C, the isolation frequency of *Escherichia coli* notably decreased. We have also found evidence in previous studies that *Escherichia coli* transcribes better at low temperatures (25°C), while its transcription and translation efficiency decreases at high temperatures (42°C) (Grill et al., 2002).

A study investigating the relationship between clinical isolates in German ICUs and ambient temperature found an increase in Gram-negative bacteria during the summer compared to the winter, while Gram-positive bacteria showed a decrease (Schwab et al., 2014). Similarly, in our study, under conditions of elevated core body temperature, we observed a higher prevalence of *Pseudomonas aeruginosa* and *Stenotrophomonas maltophilia* in the lower respiratory tract microbiota. However, the Gram-positive bacteria *Staphylococcus aureus* exhibited a higher prevalence, while the commonly ICU pathogen *Streptococcus pneumoniae* demonstrated a noticeable decrease in isolation frequency. These findings align with a published article highlighting the characteristics of *Streptococcus pneumoniae (* Schwab et al., 2014), which indicated that its activity significantly diminishes as environmental temperatures rise.

Notably, six cases of *Lactobacillus fermentum* were isolated from the lower airways of patients with heatstroke, and all six were isolated within the early days (48 hours) of admission to the ICU and only one was associated with hyperthermic resistant. *Lactobacillus fermentum* is not frequently isolated in the ICU and is more often reported as a probiotic. Supplementation with *Lactobacillus fermentum* is often beneficial to the homeostasis of the patient’s intestinal flora and to the pulmonary flora via the pulmonary-intestinal axis (Wang et al., 2022). However, there are also previous studies that have found an increased abundance of *Lactobacillus fermentum* isolated from the lower airways of asthmatic patients (Denner et al., 2016), whether this indicates a role beyond probiotics. It remains unclear whether the presence of *Lactobacillus fermentum* has a pathological implication or if it is simply due to its preference for the temperature range conducive to its growth and activity. Additional investigation is warranted to better understand the role and potential implications of *Lactobacillus fermentum* in the context of heatstroke and respiratory infections.

## Study limitations

Several limitations should be acknowledged in this study. Firstly, the sample size was relatively small, comprising only a specific population of heatstroke patients in only one geographic area (within Sichuan Province), and given the existence of pathogens that may be characterized differently in different geographic locations, a single-area study may have some limitations. Therefore, the generalizability of the results may be limited. Secondly, the absence of 16s sequencing analysis in our study limits the ability to accurately characterize the specific strains and diversity of the microbiome in the lower respiratory tract of heatstroke patients. Future prospective investigations incorporating 16s sequencing will provide a more precise understanding of heatstroke. Thirdly, although most of the isolated bacteria we described are pathogenic bacteria, it did not rule out colonizing bacteria, and it is not clear to distinguish, but this is indeed a challenge in the respiratory infection field. Fourth, given that this study is a retrospective analysis, we were unable to collect data on the bacterial load of patients, which may be a major concern for patients with severe heat stroke, this is one of the limitations of our study. Fifth, the absence of data on antibiotic resistant, due to its omission during the initial data collection phase, could be a potential reason for the persistent hyperthermia in heatstroke patients and represents a limitation of our study.

## Conclusion

Our study found that lower respiratory tract infection was common in critically ill patients with heatstroke and their epidemic pathogens had temperature-dependent behavior. These findings have significant implications for therapeutic and clinical management, especially provide a foundation for future empirical treatment strategies in heatstroke.

## Data Availability

The raw data supporting the conclusions of this article will be made available by the authors, without undue reservation.

## References

[B1] Bouchama A. Abuyassin B. Lehe C. Laitano O. Jay O. O’Connor F. G.. (2022). Classic and exertional heatstroke. Nat. Rev. Dis. Primers 8, 8. doi: 10.1038/s41572-021-00334-6 35115565

[B2] Chen H. Zhao L. Cheng L. Zhang Y. Wang H. Gu K.. (2022). Projections of heatwave-attributable mortality under climate change and future population scenarios in China. Lancet Reg. Health West Pac 28, 100582. doi: 10.1016/j.lanwpc.2022.100582 36105236 PMC9465423

[B3] Coleman J. (2022). Climate change made South Asian heatwave 30 times more likely. Nature. doi: 10.1038/d41586-022-01444-1 35606438

[B4] Denner D. R. Sangwan N. Becker J. B. Hogarth D. K. Oldham J. Castillo J.. (2016). Corticosteroid therapy and airflow obstruction influence the bronchial microbiome, which is distinct from that of bronchoalveolar lavage in asthmatic airways. J. Allergy Clin. Immunol. 137, 1398–1405.e1393. doi: 10.1016/j.jaci.2015.10.017 26627545 PMC4860110

[B5] Ding X. Liang H. Qi X. Sun G. Cheng M. Feng M.. (2023). Changes of Klebsiella pneumoniae infection and carbapenem resistance in ICU elderly infected patients before and after the COVID-19 pandemic in Zhengzhou, China. J. Infect. 86, 256–308. doi: 10.1016/j.jinf.2023.01.008 PMC9827739 36632942

[B6] Epstein Y. Yanovich R. (2019). Heatstroke. N Engl. J. Med. 380, 2449–2459. doi: 10.1056/NEJMra1810762 31216400

[B7] Feng X. Wei S. Wang S. (2020). Temperature inversions in the atmospheric boundary layer and lower troposphere over the Sichuan Basin, China: Climatology and impacts on air pollution. Sci. Total Environ. 726, 138579. doi: 10.1016/j.scitotenv.2020.138579 32305769

[B8] Folic M. M. Djordjevic Z. Folic N. Radojevic M. Z. Jankovic S. M. (2021). Epidemiology and risk factors for healthcare-associated infections caused by Pseudomonas aeruginosa. J. Chemother. 33, 294–301. doi: 10.1080/1120009X.2020.1823679 32996875

[B9] Grill S. Moll I. Giuliodori A. M. Gualerzi C. O. Bläsi U. (2002). Temperature-dependent translation of leaderless and canonical mRNAs in Escherichia coli. FEMS Microbiol. Lett. 211, 161–167. doi: 10.1111/j.1574-6968.2002.tb11219.x 12076807

[B10] Huus K. E. Ley R. E. (2021). Blowing hot and cold: body temperature and the microbiome. mSystems 6, e0070721. doi: 10.1128/mSystems.00707-21 34581596 PMC8552956

[B11] Hylander B. L. Repasky E. A. (2019). Temperature as a modulator of the gut microbiome: what are the implications and opportunities for thermal medicine? Int. J. Hyperthermia 36, 83–89. doi: 10.1080/02656736.2019.1647356 31795833 PMC6897310

[B12] Jaroszewski D. E. Webb B. J. Leslie K. O. (2012). Diagnosis and management of lung infections. Thorac. Surg. Clin. 22, 301–324. doi: 10.1016/j.thorsurg.2012.05.002 22789595 PMC7106184

[B13] Niven D. J. Laupland K. B. (2016). Pyrexia: aetiology in the ICU. Crit. Care 20, 247. doi: 10.1186/s13054-016-1406-2 27581757 PMC5007859

[B14] Paling F. P. Hazard D. Bonten M. J. M. Goossens H. Jafri H. S. Malhotra-Kumar S.. (2020). Association of staphylococcus aureus colonization and pneumonia in the intensive care unit. JAMA Netw. Open 3, e2012741. doi: 10.1001/jamanetworkopen.2020.12741 32997125 PMC7527877

[B15] Patel J. Boyer N. Mensah K. Haider S. Gibson O. Martin D.. (2023). Critical illness aspects of heatstroke: A hot topic. J. Intensive Care Soc. 24, 206–214. doi: 10.1177/17511437221148922 37260431 PMC10227888

[B16] Périard J. D. DeGroot D. Jay O. (2022). Exertional heat stroke in sport and the military: epidemiology and mitigation. Exp. Physiol. 107, 1111–1121. doi: 10.1113/eph.v107.10 36039024 PMC9826288

[B17] Schwab F. Gastmeier P. Meyer E. (2014). The warmer the weather, the more gram-negative bacteria - impact of temperature on clinical isolates in intensive care units. PloS One 9, e91105. doi: 10.1371/journal.pone.0091105 24599500 PMC3944990

[B18] Tuholske C. Caylor K. Funk C. Verdin A. Sweeney S. Grace K.. (2021). Global urban population exposure to extreme heat. Proc. Natl. Acad. Sci. U.S.A. 118, (41). doi: 10.1073/pnas.2024792118 PMC8521713 34607944

[B19] Wang W. Li Y. Han G. Li A. Kong X. (2022). Lactobacillus fermentum CECT5716 alleviates the inflammatory response in asthma by regulating TLR2/TLR4 expression. Front. Nutr. 9, 931427. doi: 10.3389/fnut.2022.931427 35911120 PMC9331901

[B20] Watts N. Amann M. Arnell N. Ayeb-Karlsson S. Beagley J. Belesova K.. (2021). The 2020 report of The Lancet Countdown on health and climate change: responding to converging crises. Lancet 397, 129–170. doi: 10.1016/S0140-6736(20)32290-X 33278353 PMC7616803

